# A new genus and species of feather duster worm (Annelida, Sabellidae) from shallow hydrocarbon seeps in the Gulf of Mexico

**DOI:** 10.3897/BDJ.13.e139552

**Published:** 2025-03-18

**Authors:** Lauren N. Rice, María Ana Tovar-Hernández, Christina I. Ellison, Craig M. Young

**Affiliations:** 1 University of Oregon, Oregon Institute of Marine Biology, Charleston, OR, United States of America University of Oregon, Oregon Institute of Marine Biology Charleston, OR United States of America; 2 Universidad Autónoma de Nuevo León, San Nicolás de Los Garza, Nuevo León, Mexico Universidad Autónoma de Nuevo León San Nicolás de Los Garza, Nuevo León Mexico

**Keywords:** Upper Louisiana Slope, methane seep, Sabellid, gregarious, epibiont, carbonate, file clam

## Abstract

**Background:**

Sabellid species are known to have a broad depth distribution and have been reported from various deep-sea habitats, including chemosynthetic systems. Despite this presence, only two species have been identified from deep water chemosynthetic habitats and only one has been identified to species. When examining hydrocarbon seep systems along the Upper Louisiana Slope in the Gulf of Mexico, we observed an abundant sabellid species new to science. The characters for the collected specimens did not match any existing genus.

**New information:**

The description for the new sabellid genus and species are presented, supported by external morphology and DNA sequence data (cytochrome c oxidase I). *Seepicolaviridiplumi* gen. nov., sp. nov. are gregarious, facultative hyper-epibionts within the examined methane seep communities and, seemingly, have a blend of morphological features of the genera *Perkinsiana* and *Pseudopotamilla*. Specimens also have several distinctive characters including the presence of a pair of peristomial chambers between the ventral lappets and parallel lamellae and the short, button-like shape of the radiolar tips. Abundance estimates for *S.viridiplumi* gen. nov., sp. nov. within the seep habitats are also presented.

## Introduction

Polychaetes in the family Sabellidae Latreille 1825 are diverse with 512 named species across 42 genera (Capa et al. 2021a). Sabellids are also recognised as ubiquitous, with individuals appearing in a variety of marine habitats, although most have been reported from shallow-water systems (reviewed by Capa et al. (2019)). However, sabellids have been reported to depths of 9735 m (Levenstein 1961). An understanding of sabellid diversity in deep-water habitats is currently limited, with a small number of reports identifying specimens to genus and others identifying only to family (Capa et al. 2021a).

Only two members of sabellid species have been identified from deep-sea chemosynthetic habitats, both belonging to *Bispira* Kröyer, 1856. *Bispirawireni* (Johansson, 1922) was described from hydrothermal vent systems in the Okinawa Trough off southern Japan at a depth of 1335 m (Capa et al. 2013). The second is an undescribed species of *Bispira* found at methane seeps from Jaco Scar, Costa Rica at depths of 1768 to 1887 m and is a newly-identified example of chemosynthetic bacterial symbiosis, where strains of methanotrophic Methylococcales bacteria were embedded in the cuticle of the radioles (Goffredi et al. 2020).

In the present study, we report findings of a third sabellid species observed at chemosynthetic systems. This species is found in hydrocarbon seeps on the Upper Louisiana Slope in the northern Gulf of Mexico, at depths between 562 and 651 m. Individuals are facultative, hyper-epibionts and can be found burrowed into the valves of *Acestaoophaga* Järnegren et al. 2007, a species of file clam and within authigenic carbonate. These worms have a combination of morphological features like those described amongst members of *Perkinsiana* Knight-Jones 1983 and *Pseudopotamilla* Bush 1905, but not *Bispira*. The species described here also has several unique morphological features, allowing for establishment of a new generic hypothesis. These findings were also supported by analysis of DNA sequence data (cytochrome c oxidase I).

## Materials and methods

### Sample collection

Specimens were collected from the hydrocarbon seeps Bush Hill, Green Canyon 234 and Brine Pool NR-1, using either ROV *Jason* deployed from the R/V *Thomas G. Thompson* (expedition TN391) or HOV *Alvin* deployed from the R/V *Atlantis* (expedition AT50-04). Collections occurred in June 2021 and October 2022, respectively. On each collection, individuals from both authigenic carbonate and *Acestaoophaga* valves were gathered using the vehicle manipulator arm and subsequently transported to the surface in insulated collection boxes. After recovery to the ship, carbonate slabs were broken into smaller pieces using a hammer and chisel and sabellids were isolated from both substrate types using dental picks. Samples were then fixed in buffered seawater-formalin, washed in fresh water and stored in 70% ethanol. Tissues used for genetic work were frozen at sea to -80^o^C. Tissue samples from two additional sabellid individuals were post-fixed in osmium tetroxide for two hours before being dehydrated in a graded ethanol series ending in two 100% ethanol baths for 20-minutes each. The tissues were then critical-point dried with CO_2_, mounted on stubs and coated with 20 nm of gold. The stubs were viewed using a Tescan Vega II SBU and ZEISS Ultra-55 scanning electron microscope.

The description is based on the holotype, with variation amongst paratypes indicated in parentheses. The thoracic glandular pattern was described by staining specimens with methyl green.

### Species abundance estimates

Observations of sabellid abundance were quantitatively examined from video footage from either the ROV *Jason* in June 2021 or the HOV *Alvin* in October 2022. Representative image frames were randomly selected for analysis from video footage of sabellid collections. As the primary mission for the dives was to conduct sample collections and to deploy scientific instrumentation, clear and continuous footage of the seafloor was scattered. Additionally, scaling lasers were haphazardly enabled throughout the dives and were often not visible in image frames. Thus, the visible surface area for each frame (the area that was well lit and in focus) was estimated using a scaling approach, based on observable equipment present in the image. The equipment used had known dimensions and helped to ensure consistent and accurate scaling. Visible sabellid tubes within each frame were counted using ImageJ (NIH). Epibiotic sabellid density was measured by counting the number of tubes present on each valve of host *Acestaoophaga*. All collected *A.oophaga* had their shell length and width recorded.

### DNA sequence analysis

Genomic DNA was extracted from each specimen using the DNEasy Blood and Tissue Kit (Qiagen) following the manufacturer's protocol. The Folmer region of the mitochondrial protein-coding gene cytocrome c oxidase I was amplified from each individual and sequenced using universal primers LCO1490 5' GGTCAACAAATCATAAAGATATTGG and HCO2198 5' TAAACTTCAGGGTGACCAAAAAATCA (Folmer et al. 1994). Each PCR was performed in a 20 µl volume using 2 µl of unquantified DNA extract, 200 µM of dNTPs (NE Biolabs), 500 nM of each primer (IDT) and 1 unit of Go Taq polymerase with supplied buffer (Promega). We used the following thermocycling profile: initial denaturation at 95^o^C for 2 min, followed by 35 cycles of: 1) denaturation, 95^o^C, 40 sec; 2) primer annealing, 45^o^C, 40 sec; 3) primer extension, 72^o^C, 1 min, followed by final extension at 72^o^C for 2 min. PCR products were verified with gel electrophoresis and those producing single bright bands of expected size were purified using SV Wizard Gel and PCR cleanup kit (Promega) according to the manufacturer's protocol. PCR products were sequenced at Sequetech (Mountain View, CA) in both directions using PCR primers.

Sequence analysis and tree-building were conducted using Geneious Prime v. 2025.0.3 (Biomatters). Sequences with a low percentage of high-quality bases (< 50%) were excluded from subsequent analysis. For included sequences, PCR primers and low-quality end-regions were trimmed off, opposing strands aligned and disagreements between them manually resolved to produce a consensus sequence. Nucleotide bases with combined PHRED score of < 20 were converted into "N"s in the consensus sequence or trimmed off. Each consensus sequence was then translated into amino acids using the Invertebrate Mitochondrial code (translation table 5) to check for the presence of stop codons.

NCBI nucleotide BLAST searches (GenBank) were used to screen for contamination and aid in specimen identification. The top 20 BLAST matches (based on pairwise identity) were downloaded and included in subsequent analysis. We specifically included available sequences of *Perkinsiana* and *Pseudopotamilla* due to morphological features shared by individuals analysed in this study, both of which are represented by type species (*Pseudopotamillareniformis* (Bruguière, 1789) and *Perkinsianafonticula* (Hoagland, 1919)) from their natural range of distribution. We included additional reference sequences (Suppl. material 1) to better contextualise the novel sequence data within Amphiglenini tribe *sensu* Tilic et al. (2020), to rule out affiliation with previously established genera and test whether the new genus may belong to a strongly supported clade (but see DNA sequence analysis below). Aside from sequences of the new species, the dataset was pared to retain a single sequence per species.

The 48 sequences in the dataset were aligned using the MAFFT plug-in using default parameters. The alignment was visually inspected for gaps and irregularities, trimmed to 661 bp (sequences of *Acromegalomma* Gil & Nishi, 2017 introduced a three bp gap relative to remaining sequences) and used to construct a Maximum Likelihood tree using RAxML (GTR+G+I substitution model with 1,000 boostraps).

Newly-generated sequences and raw trace files have been deposited in Barcode of Life Datasystems (BOLD) and GenBank (Table 1). DNA extracts are held at the Oregon Institute of Marine Biology.

## Data resources

Type specimens were deposited in the following collections: Colección Poliquetológica de la Universidad Autónoma de Nuevo León (UANL) and Colección Nacional de Anélidos Poliquetos de México, Instituto de Ciencias del Mar y Limnología, Universidad Nacional Autónoma de México (CNAP–ICML, UNAM). SEM stubs of paratypes were deposited in the Colección Nacional de Anélidos Poliquetos de México (MEB-POP-72-0058).

## Taxon treatments

### Seepicola

Rice, Tovar-Hernández, Ellison & Young gen. nov.

549C58C1-827C-5B95-91A0-FABD358960E3

71A03636-1E5F-4734-85F1-C69906B4D666

Seepicola viridiplumi Rice, Tovar-Hernández, Ellison & Young, 2025, gen. nov. designated by monotypy.. Type species.

#### Description

Medium size species in the subfamily Myxicolinae with a moderate number of radioles arranged in semi-circular radiolar lobes. Brachial crown symmetrical, with all radioles nearly the same length. Brachial crown with basal, dorsal, and ventral flanges. Palmate membrane, radiolar flanges and radiolar eyes absent. Pinnules paired. Radiolar tips entire (unbranched). Dorsal lips with mid rib (radiolar appendages) and dorsal pinnular appendages. Ventral lips and parallel lamellae present. Ventral sacs absent. Ventral peristomial chambers present, located between the ventral lappets and parallel lamellae. Peristomial loops absent. Peristomial eyes present. Collar chaete narrowly hooded. Glandular ridge on chaetiger 2 absent. Ventral shields are well differentiated. Thoracic chaetae with a superior group of narrowly hooded chaete, inferior group with paleate chaetae. Neurochaetae with tear-drop shaped companion chaetae and avicular uncini. Teeth above the main fan of equal size, hood absent, breast well-developed, expanded, handles of medium length. Interamal eyespots absent. Abdominal chaetae elongate, broadly hooded. Abdominal avicular uncini with teeth above the main fang of equal size, breast well developed, short handled. Pygidium without anal cirrus. Pygidial eyes absent.

#### Etymology

The genus name refers to the fact that the type species was found in hydrocarbon seep habitats (Seeps, combined with the latin -*cola* = 'dweller'). It should be regarded as an invariant compound noun in apposition (Read et al. 2017; pg. 19).

### Seepicola viridiplumi

Rice, Tovar-Hernández, Ellison & Young sp. nov.

9187DDB0-EB80-57FB-8B38-38312388CAD6

78FAA822-18F2-4D0E-A31C-FA223BC469E6

#### Materials

- **Type status:** Holotype. **Occurrence:** occurrenceID: 9F20A7F4-BA4E-5B6A-B41E-DFB18FD5D47E; **Record Level:** type: PhysicalObject; collectionID: BHSa144; institutionCode: CNAP–ICML, UNAM; ownerInstitutionCode: POH-72-001; basisOfRecord: PreservedSpecimen; dynamicProperties: Cruise Code = TN391, Date of Collection = 14 June 2021, Latitude = 27.78237117, Longitude = -91.50830855, Depth = 562 m, Notes = epibiotic on *Acestaoophaga*
- **Type status:** Paratype. **Occurrence:** occurrenceID: 3A8CBE5B-263B-5D5B-AFC7-A7BA2EB395E3; **Record Level:** type: PhysicalObject; collectionID: BPSa115; institutionCode: CNAP–ICML, UNAM; ownerInstitutionCode: POP-72-003; basisOfRecord: PreservedSpecimen; dynamicProperties: Cruise Code = AT50-04, Date of Collection = 16 October 2022, Latitude = 27.723677, Longitude = -91.279372, Depth = 651 m, Notes = epibiotic on *Acestaoophaga*
- **Type status:** Paratype. **Occurrence:** occurrenceID: AB4A10DA-E875-5DD1-BF99-6023F33E97E8; **Record Level:** type: PhysicalObject; collectionID: BHSa167; institutionCode: CNAP–ICML, UNAM; ownerInstitutionCode: POP-72-004; basisOfRecord: PreservedSpecimen; dynamicProperties: Cruise Code = TN391, Date of Collection = 14 June 2021, Latitude = 27.78237117, Longitude = -91.50830855, Depth = 562 m, Notes = epibiotic on *Acestaoophaga*
- **Type status:** Paratype. **Occurrence:** occurrenceID: 46A27349-00EC-53A0-8824-CDC03618D18D; **Record Level:** type: Physical Object; collectionID: BPSa92; institutionCode: UANL; ownerInstitutionCode: UANL-8188; basisOfRecord: PreservedSpecimen; dynamicProperties: Cruise Code = AT50-04, Date of Collection = 16 October 2022, Latitude = 27.723677, Longitude = -91.279372, Depth = 651 m, Notes = epibiotic on *Acestaoophaga*
- **Type status:** Paratype. **Occurrence:** occurrenceID: D8989B89-32DD-5A42-82F1-CAF2907013C2; **Record Level:** collectionID: BHSa127; institutionCode: UNAL; ownerInstitutionCode: UANL-8189; basisOfRecord: PreservedSpecimen; dynamicProperties: Cruise Code = AT50-04, Date of Collection = 15 October 2022, Latitude = 27.723677, Longitude = -91.279372, Depth = 562 m, Notes = free-living in authigenic carbonate
- **Type status:** Paratype. **Occurrence:** occurrenceID: D7E69433-518E-5E1B-9CC5-82277F14B884; **Record Level:** type: PhysicalObject; collectionID: BPSa88; institutionCode: CNAP–ICML, UNAM; ownerInstitutionCode: MEB-POP-72-0058; basisOfRecord: SEM Stub; dynamicProperties: Stub Numbers = 1 - 3, Cruise Code = AT50-04, Date of Collection = 16 OCtober 2022, Latitude = 27.723677, Longitude = -91.279372, Depth = 651, Notes = epibiotic on *Acestaoophaga*
- **Type status:** Paratype. **Occurrence:** occurrenceID: CD767440-FF78-5D67-AD62-33E69A9830C9; **Record Level:** type: PhysicalObject; collectionID: BPSa123; institutionCode: CNAP–ICML, UNAM; ownerInstitutionCode: MEB-POP-72-0058; basisOfRecord: SEM Stub; dynamicProperties: Stub Numbers = 4 & 5, Cruise Code = AT50-04, Date of Collection = 16 October 2022, Latitude = 27.723677, Longitude = -91.279372, Depth = 651 m, Notes = epibiotic on *Acestaoophaga*

#### Description

**Figure 1.**
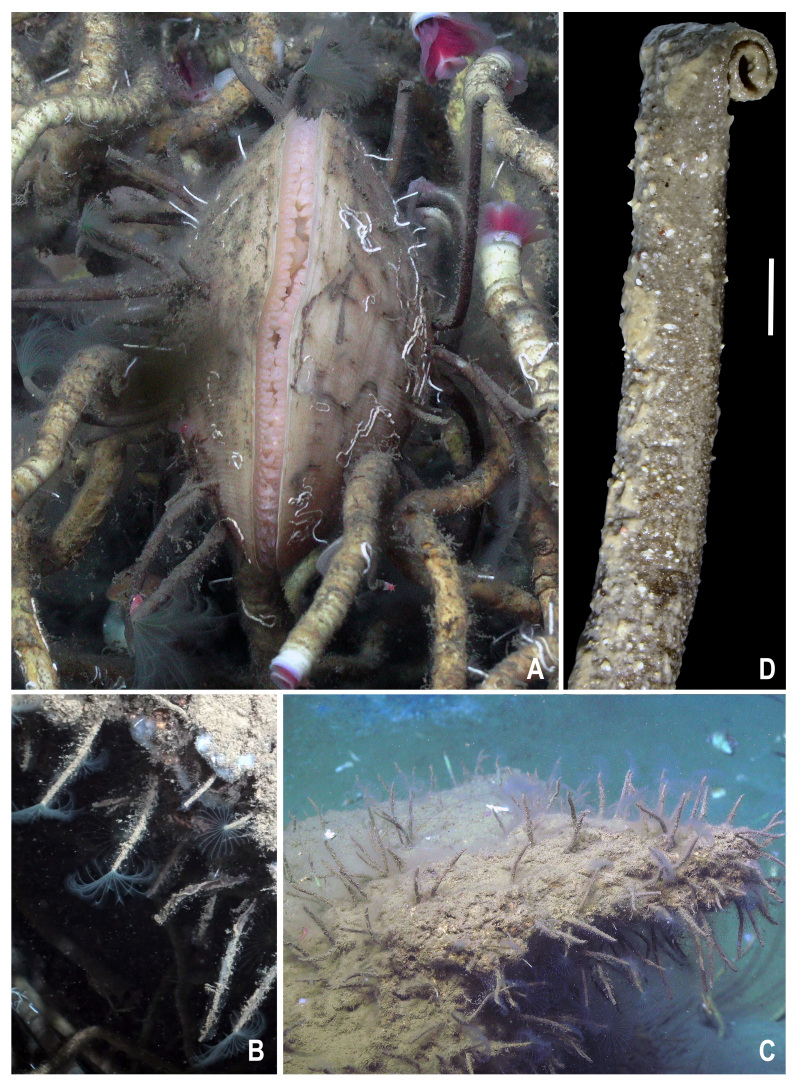
*In situ* images of *Seepicolaviridiplumi* gen. nov., sp. nov. **A** Epibiotic individuals on a host *Acestaoophaga* file clam. Red-pink plumes belong to the siboglinid tube worm *Lamellibrachialuymesi*; **B** Close-up of individuals and branchial crowns on authigenic carbonate; **C** Aggregate of individuals on authigenic carbonate; **D** Tube of *S.viridiplumi* gen. nov., sp. nov. with a muddy outer lining and curled distal end. Scale bar represents 2 mm.

Tubes composed of fine sand and distal end tightly curled (Fig. 1D). Branchial crown light green (Fig. 1) and trunk cream to red-brown colour in live worms. Preserved specimens with dark to pale cream-coloured trunk and branchial crown whitish. Branchial crown length 7.2 mm (4-7.5 mm) with 12 pairs of radioles (same for paratypes). Trunk length 39 mm (28-32 mm), 2.5 mm wide (1.4-2 mm). Fifteen thoracic (13-18) and 121 abdominal chaetigers (97-143).

**Figure 2.**
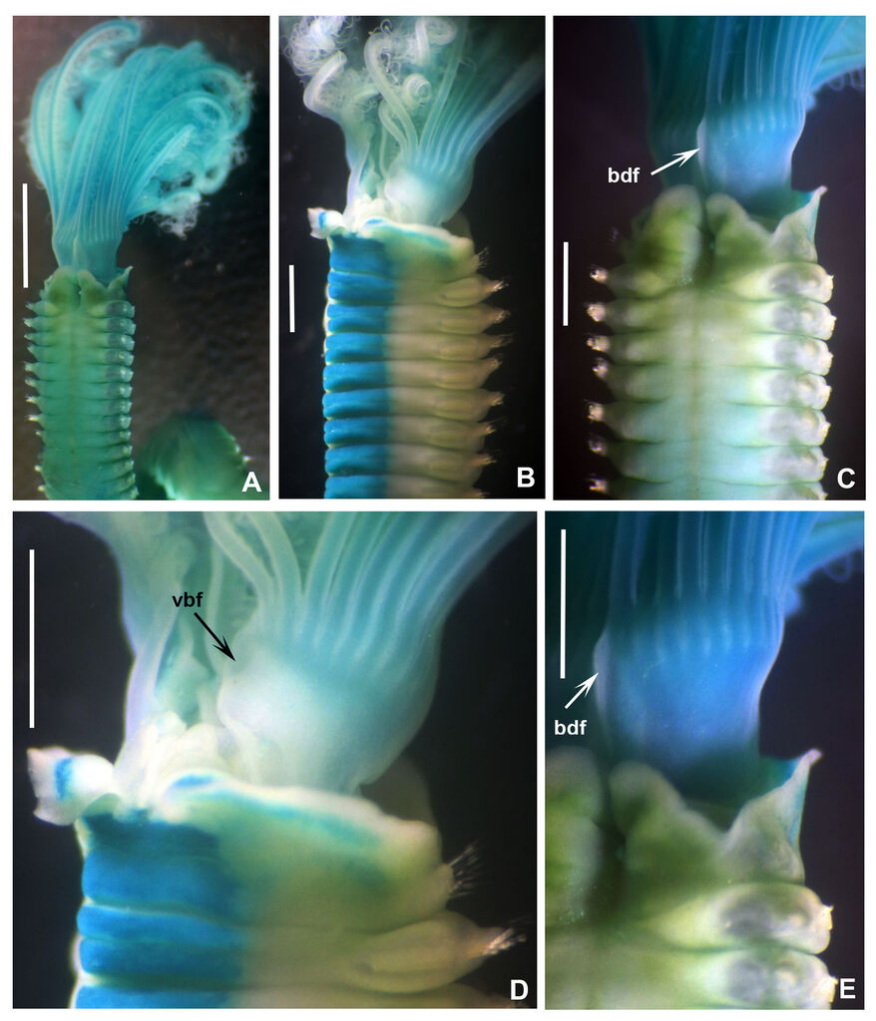
*Seepicolaviridiplumi* gen. nov., sp. nov. stained with methyl green. **A** Radiolar crown and thorax, dorsal view; **B** Thorax and base of radiolar crown, ventrolateral view; **C** Thorax and base of radiolar crown, dorsal view; **D** Detail of ventral branchial lobe flange; **E** Detail of dorsal branchial lobe flange. A-E) Holotype (CNAP–ICML, UNAM, POH-72-001). Abbreviations: bdf = basal dorsal flange, vbf = ventral basal flange. Scale bars: A) 1 mm, B-E) 0.8 mm.

**Figure 3.**
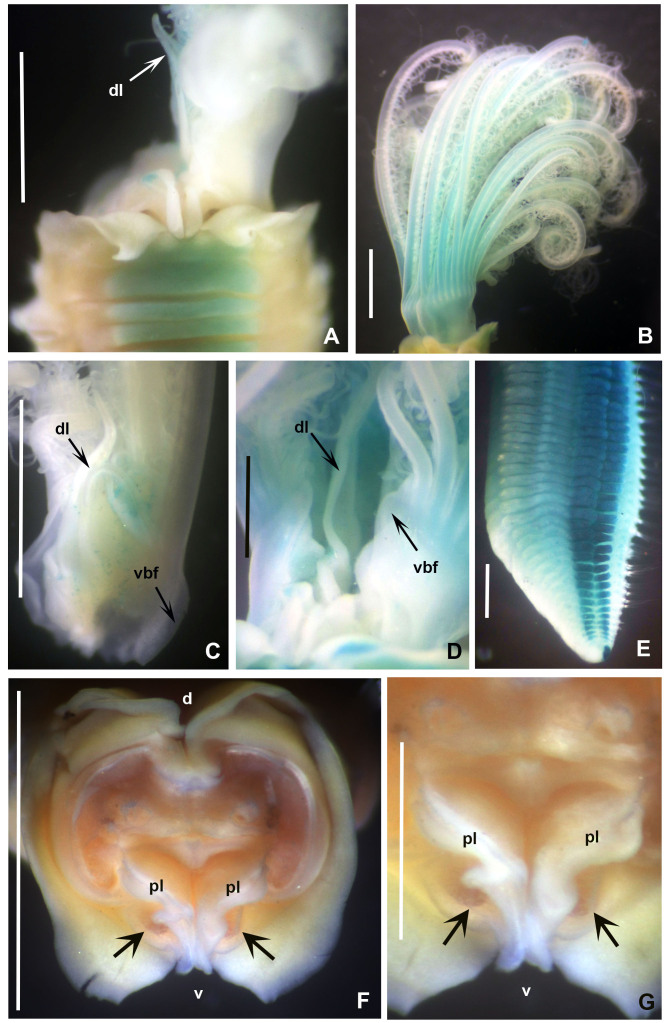
*Seepicolaviridiplumi* gen. nov., sp. nov. stained with methyl green. **A** Collar, ventral view, right radiolar lobe removed; **B** Radiolar crown, lateral view; **C** Inner margin of right branchial lobe; **D** Detail of dorsal lip and ventral branchial lobe flange; **E** Posterior end; **F** Peristomium, frontal view, radiolar crown removed, peristomial chambers indicated with arrows; **G** Detail of mouth and peristomial chambers pointed with arrows. A, C, F-G) Paratype 3, B, D-E) Holotype (CNAP–ICML, UNAM, POH-72-001). Abbreviations: d = dorsal, dl = dorsal lip, pl = parallel lamella, v = ventral, vbf = ventral basal flange. Scale bars: A-C) 1 mm, D-E, G) 0.5 mm, F) 1.5 mm.

**Figure 4.**
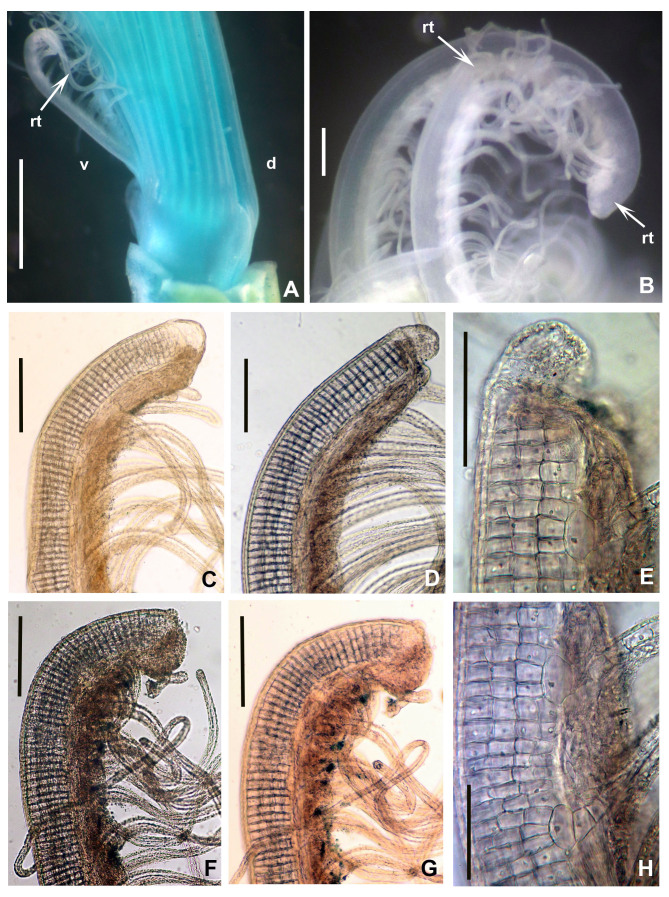
*Seepicolaviridiplumi* gen. nov., sp. nov. **A** Proximal end of branchial crown, lateral view, stained with methyl green (Paratype 2). **B** Radiolar tips; **C–G** Details of the radiolar tips showing cartilaginous skeletal cells and massive tissue on inner margins of radioles; **H** Detail of radiolar and pinnular skeletal cells. A) Holotype (CNAP–ICML, UNAM, POH-72-001), B-H) Paratype 1 (CNAP–ICML, UNAM, POP-72-003). Abbreviations: d = dorsal, v = ventral, rt = radiolar tips. Scale bars: A) 1 mm, B-G) 150 μm, H) 75 μm.

Branchial crown is mostly symmetrical (Fig. 3B) with 2-3 ventral-most radioles with long, filiform tips as long as the space of 12 pinnules (Fig. 4A). Ventral-most radioles 1/8 the length of dorsal radioles. Branchial lobes short, as long as the peristomium collar segment (Fig. 2A-C). Mid-dorsal radiole lobe basal flanges narrow, entire and translucent (Fig. 2C and E; Fig. 4A). Ventral radiole lobe flanges short, broad, with rounded margin, transluscent (Fig. 2D, Fig. 3D and Fig. 4A). Radiolar flanges and palmate membrane absent (Fig. 2D). Pinnules long, uniform along radioles. Radiolar eyes absent. All radioles of holotype, except ventral most, with short, broadly truncate distal ends (Fig. 4B). Inner longitudinal margins of radioles with truncate tips have thick, brownish tissue (Fig. 4C-H); this tissue broadest near radiole tips, giving appearance of radiolar eyes, but lacking ommatidia (Fig. 4D-G). Some paratypes with mixed radiolar tips (long-filiform, short buttom-like). Radiolar skeleton with four rows of cells in lateral view (Fig. 4E and H). Crown of paratype 1 (BPSa115) regenerating: shorter in comparison to holotype and other paratypes; pinnules rudimentary.

Anterior peristomial ring slightly exposed dorso-laterally between dorsal pockets and lateral collar margin (Fig. 2E; Fig. 7A). Posterior peristomial ring collar with mid-dorsal margins fused to faecal groove (Fig. 2E; Fig. 7A); dorsal collar margins form two low, rounded lappets (Fig. 2A and C). Dorso-lateral collar margins entire, forming "V" (Fig. 2A, C and E). Ventral lappets triangular with rounded distal margins mid-ventrally incised (Fig. 2D; Fig. 3A and Fig. 7A). Peristomial eyes present in paratypes 1 (BPSa115) and 2 (BHSa167). Dorsal lips triangular, erect, dorsal pinnular and radiolar appendages present (Fig. 3A, C-D and F); ventral lips short and rounded, ventral sacs absent. Peristomial chamers oval, low, translucent with internal dark brownish tissue placed between ventral lappets and parallel lamellae.

**Figure 5.**
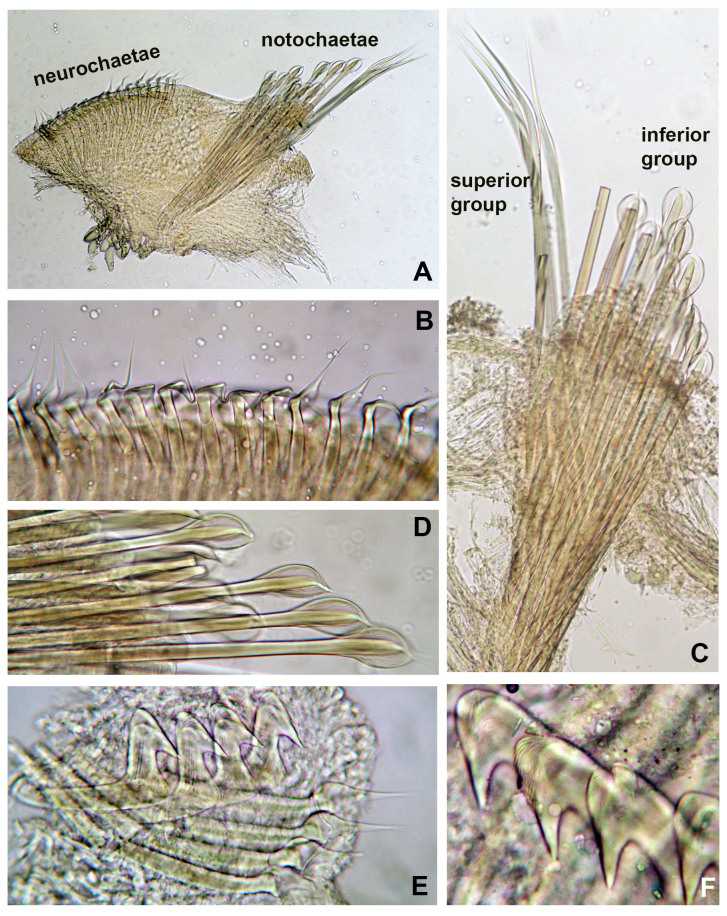
*Seepicolaviridiplumi* gen. nov., sp. nov., thoracic chaetae from chaetiger 11. **A** Thoracic noto- and neurochaetae; **B** Companion chaetae; **C** Notochaetae; **D** Inferior rows of paleate chaetae; **E** Avicular uncini and companion chaetae; **F** Detail of heads (main fangs) of uncini. A-F) Holotype (CNAP–ICML, UNAM, POH-72-001). Magnification: A) 4 x, B, D-E) 40 x, C) 10 x, F) 100 x.

**Figure 6.**
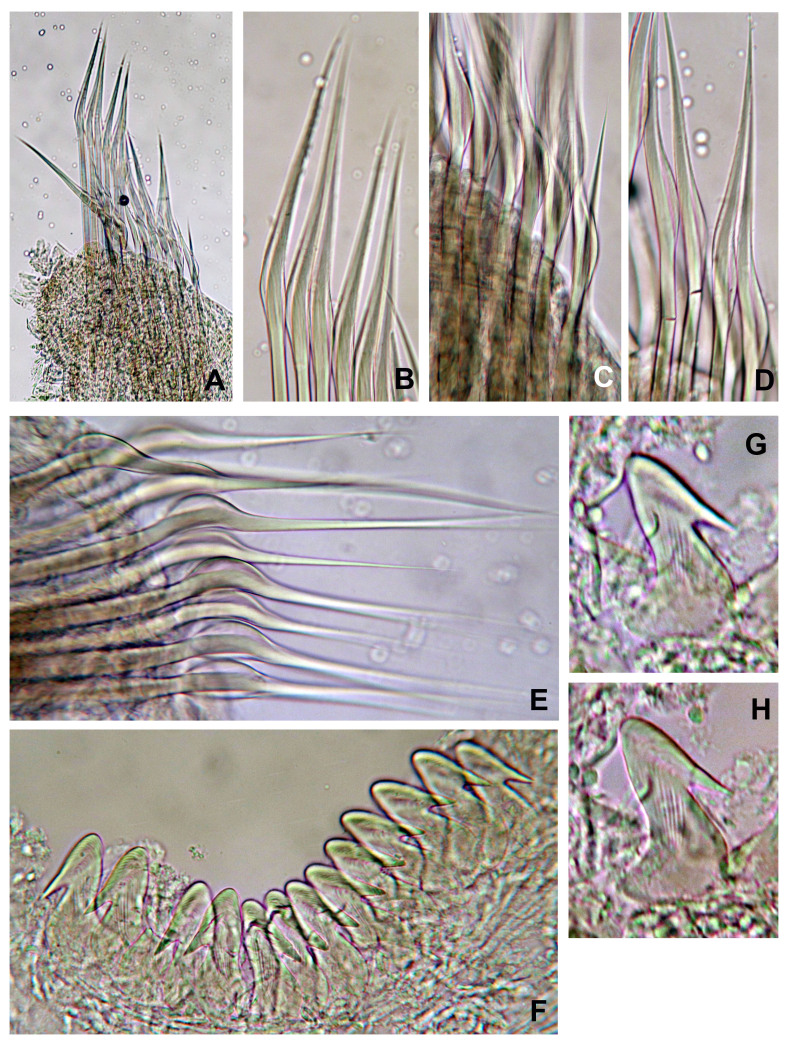
*Seepicolaviridiplumi* gen. nov., sp. nov., chaetiger 1 and abdominal chaetae. **A** Chaetiger 1; **B** Chaetiger 1 superior rows of elongate, narrowly hooded and slightly curved chaetae; **C** Chaetiger 1 inferior group composed of elongate, hooded chaete broader than the superior group; **D** Chaetiger 4 abdominal neurochaetae; **E** Chaetiger 4 broadly hooded abdominal neurochaete; **F** Avicular uncini from chaetiger 4; **G, H** Details of uncini from chaetiger 4. A-H) Holotype (CNAP–ICML, UNAM, POH-72-001). Magnification: A) 10 x, B-H) 40 x.

**Figure 7.**
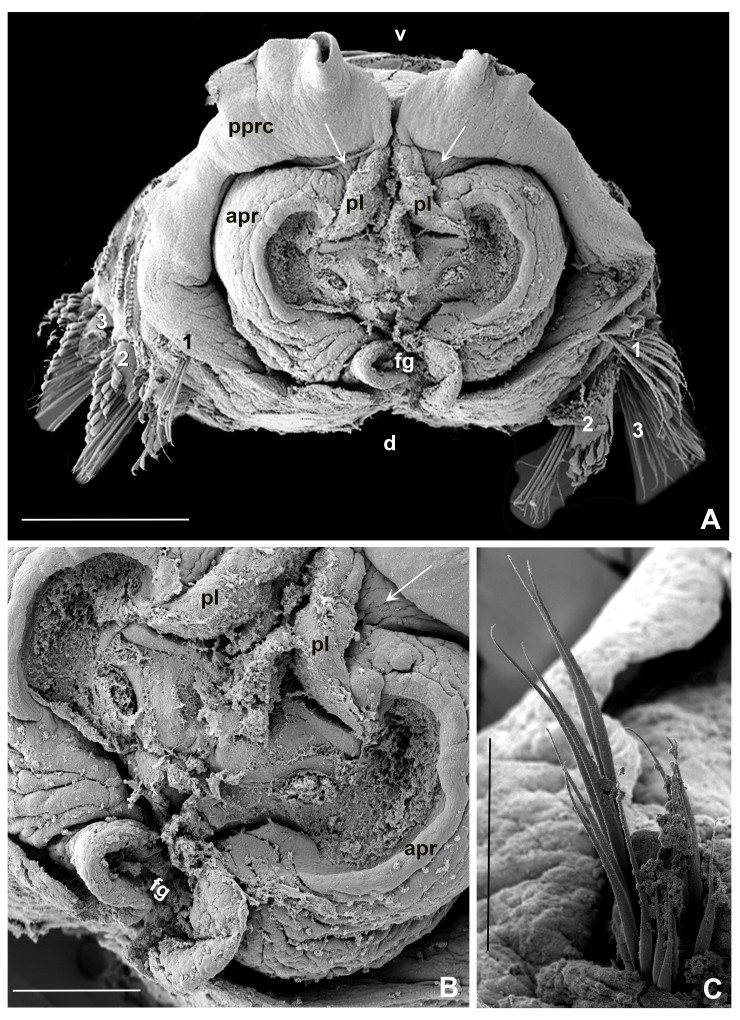
*Seepicolaviridiplumi* gen. nov., sp. nov. SEM of peristomium. **A** Frontal view of peristomium (crown removed) and peristomial chambers (arrows); **B** Detail of peristomial chambers between right ventral lappet of collar and parallel lamella; **C** Chaetiger 1 with two groups of elongate narrowly-hooded chaetae. A-B) Stub 1, C) Stub 4 (BPSa88 & BPSa123, CNAP–ICML, UNAM, MEB-POP-72-0058). Abbreviations: apr: anterior peristomial ring, d: dorsal, fg: faecal groove, pl: parallel lamellae, pprc: posterior peristomial ring collar, v: ventral, 1, 2 and 3 in A indicates number of chaetigers. Scale bars: A) 500 μm, B) 200 μm, C) 100 μm.

**Figure 8.**
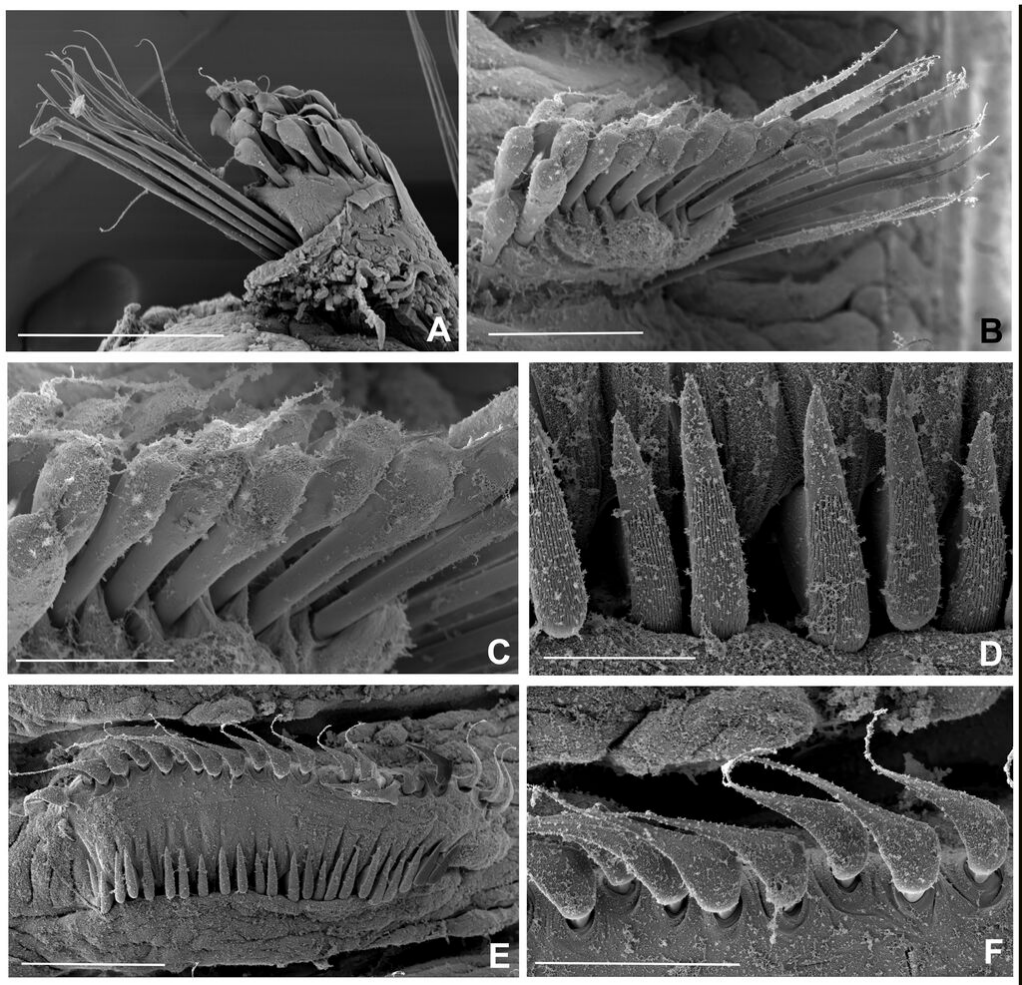
*Seepicolaviridiplumi* gen. nov., sp. nov., SEM micrographs of chaetae. **A** Chaetiger 2; **B** Chaetiger 5; **C** Detail of paleate chaetae from chaetiger 5; **D** Dentition of uncini, chaetiger 5; **E** Uncini and companion chaete, chaetiger 5; **F** Detail of companion chaetae, chaetiger 5. A) Stub 1 (BPSa88), B-C) Stub 5 and D-F) Stub 4 (BPSa123). All in CNAP–ICML, UNAM, MEM-POP-0058. Scale bars: A) 200 μm, B) 100 μm, C) 50 μm, D) 20 μm, E) 100 μm, F) 50 μm.

Chaetiger 1: (Fig. 6A-C, Fig. 7A, C), superior row long, narrowly-hooded notochaetae (Fig. 6A, B and Fig. 7C), inferior row one-half as long as superior with hoods slightly broader (Fig. 6C, Fig. 7C). Ventral shield rectangular, slightly divided transversally and longer than subsequent thoracic shields (Fig. 3A). Chaetigers 2-16 (2-13, 2-15, 2-16 or 2-18) rectangular ventral shields divided transversally in two equal parts; tori do not contact ventral shields (separated by reduced space) (Fig. 2B; Fig. 3A). Subsequent thoracic notochaetae in superior and inferior groups (Fig. 5C, Fig. 8A): superior row elongate, narrowly hooded; inferior group paleate, arranged in two rows with pointed mucro (Fig. 5D, Fig. 8A-C). Neurochaetae with companion chaetae and avicular uncini (Fig. 5A, B, Fig. 8E, F). Avicular uncini with several rows of small, similar-sized teeth above main fang (covering proximal half of main fang) (Fig. 8D), breast well developed, main fang with high crest and handles 2.5 times longer than main fang (Fig. 5E, F). Companion chaetae with symmetrical membranes teardrop-shaped (Fig. 5B, E, Fig. 8E, F) with long handles (Fig. 5A, E).

**Figure 9.**
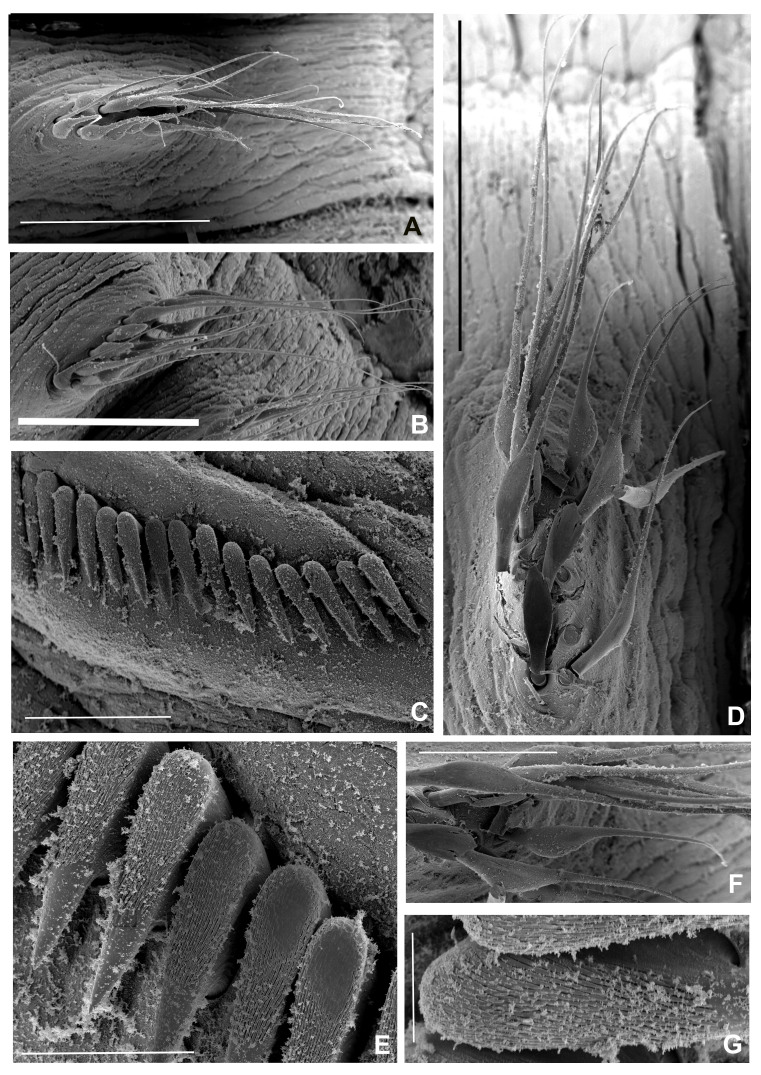
*Seepicolaviridiplumi* gen. nov., sp. nov., SEM micrographs of abdominal chaetae. **A** Anterior neuropodial fascicle, chaetiger 16; **B** Posterior neuropodial fascicle, chaeitiger 20; **C** Anterior abdominal uncini, chaetiger 16; **D** Anterior neruopodial fascicle, chaetiger 16; **E, G** Uncini dentition, chaetiger 20; **F** Detail of broadly-hooded chaetae, chaetiger 16. A-B, D, F) Stub 3 (BPSa88), C, E, G) Stub 2 (BPSa88). All in CNAP–ICML, UNAM, MEB-POP-72-0058. Scale bars: A-B, D) 200 μm, C) 50 μm, E) 20 μm, F) 100 μm, G) 8 μm.

Abdominal segments with elongate, broadly-hooded neurochaete (Fig. 6A-D, Fig. 9A), with basal, broad knee (Fig. 6E, Fig. 9F). Neurochaetae in last quarter of body two times longer than in anterior abdominal segments (Fig. 9B). Notopodial uncini avicular with several rows of teeth above main fang, extending over 3/4 of main fang (Fig. 9C, E-G). Breast well defined, handles very short, shorter than length of main fang (Fig. 6F-H). Pygidium rounded, eyes absent (Fig. 3E). Gametes not observed.

#### Etymology

The specific epithet refers to the color of the radiolar crown in living specimens, from the Latin *viridi* meaning 'green'.

#### Taxon discussion

Several morphological observations led to the specimens being placed in a monotypic genus, rather than in *Pseudopotamilla* or *Perkinsiana*. The specimens have dorsal and ventral branchial lobe flanges as in *Pseudopotamilla*. However, specimens lack compound radiolar eyes and asymmetrical radiolar crown in *Pseudopotamilla* species (Table 2). On the other hand, members of *Potamilla* Malmgren 1866, lack radiolar eyes (Knight-Jones 1983, Fitzhugh 1989). Members of *Seepicolaviridiplumi* gen. nov., sp. nov. cannot be placed in *Potamilla* because the former have dorsal lips with radiolar appendages and lack a palmate membrane, whereas *Potamilla* have dorsal lips without radiolar appendages, but a palmate membrane is present, amongst other differences (Table 2).

Fitzhugh (1989) emphasised that *Perkinsiana* Knight-Jones 1983, is not defined by any synapomorphies to indicate monophyly. Capa (2007) and Tovar-Hernández et al. (2012) both described new species of *Perkinsiana* and remarked on the existing problem of properly assigning species to this genus. In the same context, *Pseudopotamilla* is not defined by any synapomorphies (Fitzhugh 1989; Capa 2007) and, if not monophyletic, this could have distinct implications for recognising *Eudistylia* Bush, 1905 and *Schizobranchia* Bush, 1905, as the clade (*Pseudopotamilla* (*Eudistylia*, *Schizobranchia*)) is defined by the presence of branchial lobe flanges and compound radiolar eyes. In both *Pseudopotamilla* and *Perkinsiana*, species are known to burrow in hard limestone, dead coral, barnacles and mollusc shells (Chughtai and Knight-Jones 1988, Knight-Jones et al. 2017, Capa et al. 2019, Tovar-Hernández et al. 2020). There are 18 valid *Perkinsiana* species and 23 *Pseudopotamilla* species, according to Tovar-Hernández et al. (2020).

Members of *S.viridiplumi* gen. nov., sp. nov. lack a palmate membrane and radiolar flanges present in some *Perkinsiana* species. Additionally, members of *S.viridiplumi* gen. nov., sp. nov. have abdominal uncini with reduced handles, whereas these structures are short to medium length in *Perkinsiana*. Three types of abdominal chaetae can be present *Perkinsiana* species, whereas these are elongate, broadly hooded in all chaetigers of *S.viridiplumi* gen. nov., sp. nov.

Furthermore, the new species have distinctive features not previously described: the peculiar shape of radiolar tips, presence of the brown tissue on radioles and presence of a pair of peristomial chambers between the internal wall of the ventral lappets and parallel lamellae. Other features of the chaetae and uncini are typical of *Pseudopotamilla*. As with the new species, some species of *Pseudopotamilla* also have tubes that curl tightly at the end (Knight-Jones et al. 2017), but branched or bifurcated tubes, which is an indication of asexual reproduction, were not seen. However, the variable number of thoracic chaetigers observed in *S.viridiplumi* gen. nov., sp. nov. might be indicative of asexual reproduction (Kolbasova et al. 2013, Capa and Murray 2016).

## Analysis

### DNA sequence analysis

**Figure 10.**
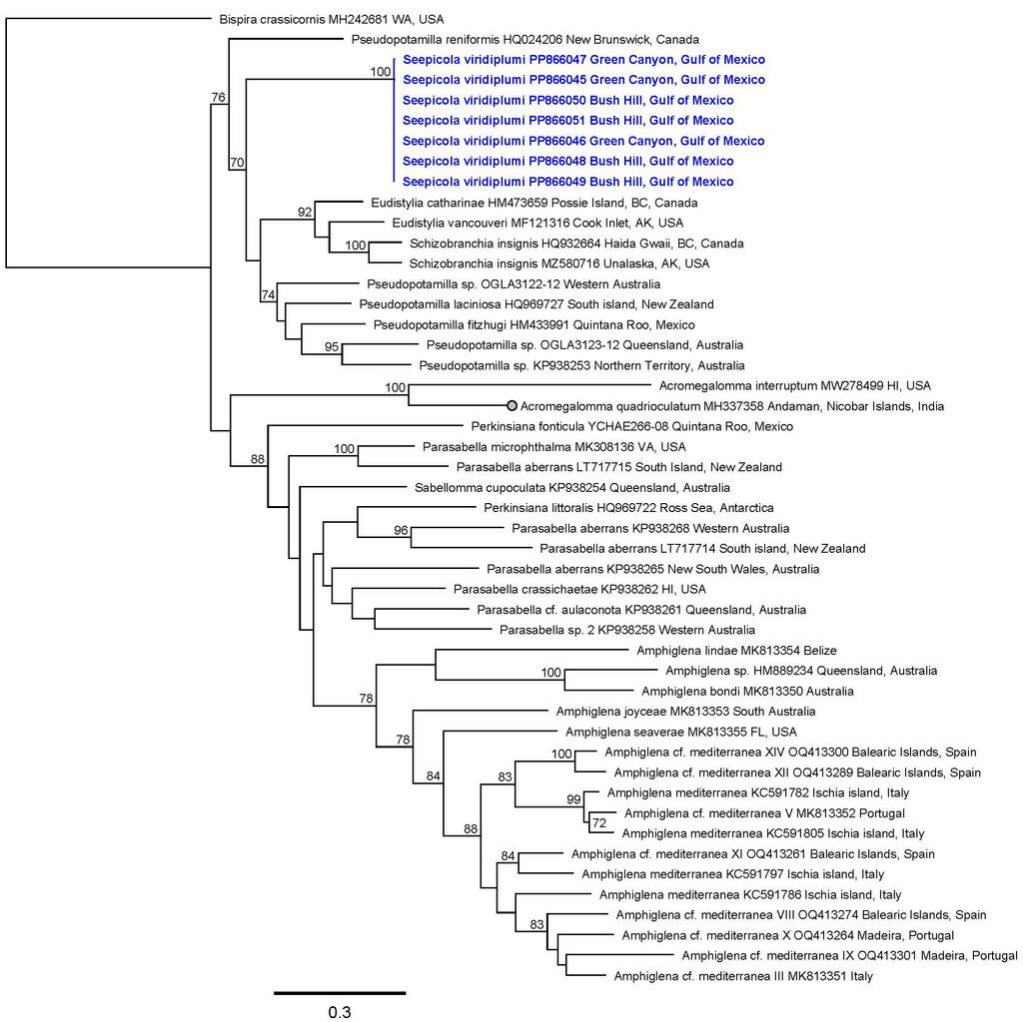
Maximum Likelihood tree (COI) showing sequences of *Seepicolaviridiplumi* gen. nov., sp. nov. in the context of those from established genera within Amphiglenini tribe *sensu* Tilic et al. (2020). Tip labels refer to, in order, species identification, GenBank accession number and collection location. Numbers refer to bootstrap support, values ≥ 70% are shown.

The sequences obtained from individuals of *Seepicolaviridiplumi* gen. nov., sp. nov. (BOLD:AFX5289) are both highly similar to each other (99.2%) and significantly divergent from reference sequences of other sabellid genera analysed in this study (23–35%). The average sequence divergence between *S.virdiplumi* gen. nov., sp. nov. and the most likely congeners, based on morphology, is 24.5–28.5% (*Pseudopotamilla* and *Perkinsiana*, respectively). Two major clades were recovered with moderate support in our analysis: one containing *Eudistylia*, *Pseudopotamilla*, *Schizobranchia* and *Seepicola* new genus (76% of boostraps) and the other, *Amphiglena* Claparède, 1864, *Parasabella* Bush, 1905, *Perkinsiana* and *Sabellomma* Nogueira, Fitzhugh & Rossi, 2010 (88%). The most strongly-supported intergeneric clade was *Eudistylia* + *Schizobranchia* (92% of bootstraps) (Fig. 10).

We cannot speak of an appropriate genus-level cut-off based on sequence similarity/divergence, as it is expected to vary significantly between taxa due to the subjectivity involved in assigning species to higher taxonomic ranks and variation in the rate of sequence evolution amongst taxa included within them. Given that rapid evolution of the third codon position in the COI barcoding region inevitably results in sequence saturation at deeper nodes, these have implications for the threshold at which this renders such sequences uninformative.

We recognise the limitations associated with the exclusive use of COI in our analysis and acknowledge that, on its own, it is not a suitable marker for credible phylogenetic inference, but point out that a robust phylogenetic analysis is beyond the scope of this paper and that the phylogeny recovered here, in which many clades are poorly supported, is not especially informative about the relationship of the new species relative to other sabellid genera. As such, we regard the main results of the genetic analysis to be: 1) sequence similarity amongst individuals of *S.viridiplumi* gen. nov., sp. nov. is indicative of an intraspecific relationship, 2) we could not identify any closely-related species in public databases that would contradict the generic hypothesis, based on the unique combination of morphological characters and 3) the placement of the new species relative to other sabellid genera is consistent with membership in tribe Amphiglenini. This latter point is strengthened when considered in conjunction with other lines of evidence, such as previous genetic studies and morphology.

The first inferred phylogenetic hypotheses to explain characters of Sabellidae proposed the grouping of *Potamilla*, *Perkinsiana*, *Potaspina*, *Pseudopotamilla*, *Eudistylia* and *Schizobranchia* within "Clade VII" based on morphology (Fitzhugh 1989). Members of "Clade VII" shared the presence of dorsal pinnular appendages and elongate, broadly-hooded chaetae in all abdominal fascicles. *Potamilla*, *Potaspina* and *Perkinsiana* formed a polytomy with *Pseudopotamilla*-*Eudistylia*-*Schizobranchia*, which was defined by the presence of unpaired compound radiolar eyes and dorsal basal flanges on the branchial crown (Fitzhugh 1989). Posteriorly, Nogueira et al. (2010), based on morphology, inferred that *Pseudopotamilla*, *Eudistylia*, *Schizobranchia* and *Sabellacerasina* form a group and *Potaspina*, *Potamilla* and *Perkinsianarubra* a polytomy, again amongst the most apomorphic genera. Then, Capa et al. (2011), using a combined analysis of morphological and molecular data, identified a "Clade IB" including *Eudistylia*, *Pseudopotamilla*, *Schizobranchia* amongst many other genera, supported by the presence of companion chaetae in the thorax and broadly-hooded abdominal chaetae. More recently, using transcriptomes, *Eudistylia* and *Pseudopotamilla* were nested within the Tribe Amphiglenini, Subfamily Myxicolinae according to Tilic et al. (2020). Under this scenario, *Seepicola* new genus would belong to the Amphiglenini Tribe, Myxicolinae subfamily.

Further works will be necessary to elucidate placement of the new genus within the broader phylogenetic context of Sabellidae, which will eventually facilitate a better understanding of character evolution.

Notwithstanding, it is important to acknowledge the utility of COI sequence data as the universal barcoding gene for most metazoan taxa (Hebert et al. 2003). As such, it is an important tool in species identification and discovery (including detection of cryptic species (Nygren 2013), diversity assessment, biomonitoring and conservation, the recognition of invasive species, an improved understanding of biogeography (Hutchings and Kupriyanova 2018), community composition, trophic relationships and dispersal potential. For these reasons, they have become an important component of integrative taxonomy (e.g. De Queiroz (2007); Capa et al. (2021b)) and inclusion of COI barcodes in species description facilitates the species incorporation into studies addressing the biological questions outlined above.

The ability to identify species with COI has become increasingly important as it underlies the efficacy of the large-scale metabarcoding studies which have become so common, but efforts to do so are routinely hindered by incomplete reference databases which are by no means representative of true diversity (e.g. Mugnai et al. (2021)). Towards the effort to increase representation of species in reference databases, especially those belonging to understudied taxa or from under-sampled habitats, it is of special importance to obtain barcodes from type material or type locality (Porter and Hajibabaei 2018; Hutchings and Kupriyanova 2018), as we have done in this study.

### Species abundance estimates

From the analysis of ROV images, sabellid density within the three seep systems averaged 330 ± 233.3 (stdev) m^-2^, but ranged from 98 to 655 individuals m^-2^. At each seep, sabellid density was most concentrated at vertical facies on authigenic carbonate outcrops and more dispersed on horizontal surfaces (Fig. 1C). Occasionally, individuals were observed on deposits of shell hash. *In situ* observations and video footage showed that aggregations at each of the collection sites were composed of specimens of varying sizes and likely belonged to younger (shorter tubes) and older (taller) individuals. Observed *S.viridiplumi* gen. nov., sp. nov. tubes emergent from the substrate ranged from approximately 4 mm to 104 mm and were intermingled within the aggregations. The aggregations at Bush Hill appeared to be more extensive than at Brine Pool NR-1 and Green Canyon 234, although this could not be quantified. This difference likely corresponds to the abundance of exposed authigenic carbonate at Bush Hill. Epibiotic *S.viridiplumi* gen. nov., sp. nov. also had varying densities on host *Acestaoophaga*. While several *A.oophaga* bore only a single sabellid, one file clam 10 cm long and 8 cm across, collected from Bush Hill, had 58 individual *S.viridiplumi* gen. nov., sp. nov.

## Discussion

*Seepicolaviridiplumi* gen. nov., sp. nov. is the third sabellid species to be identified from chemosynthetic habitats and is a species of high abundance at some of the best described hydrocarbon seeps (Cordes et al. 2009). The biology and adaptations of sabellids to deep-sea and chemosynthetic habitats is mostly unknown (Capa et al. 2021a), although they are hypothesised to act as opportunistic suspension feeders (Capa et al. 2019). However, with the identification of a chemosynthetic species of *Bispira* at methane seeps in Costa Rica (Goffredi et al. 2020), sabellid taxa have become recognised as potential hosts for chemosymbiotic bacteria (Sinner et al. 2023). The presence of thick, brown tissue along the radioles of *S.viridiplumi* gen. nov., sp. nov. could indicate a potential for symbiotic bacterial presence, although this warrants further investigation. A chemosymbiotic relationship could help explain the high abundances of *Seepicolaviridiplumi* gen. nov., sp. nov. within the three study systems. It is also likely that the species is simply utilising the prevalence of hard substrate within the seeps and the abundances could be supported by the heightened local productivity.

The facultative epibiotic nature of *S.viridiplumi* gen. nov., sp. nov. also warrants further study, as do the unquantified trophic pathways utilised by *S.viridiplumi* gen. nov., sp. nov. (Levin et al. 2016, Capa et al. 2021a). The differences in height above the seafloor between epibiotic and free-living *S.viridiplumi* gen. nov., sp. nov. may have ramifications on diet and fitness of individuals. By settling on the epizooitic *Acestaoophaga* file clams, the epibiotic sabellids are potentially exposed to stronger currents unhindered by the benthic boundary layer.

## Supplementary Material

XML Treatment for Seepicola

XML Treatment for Seepicola viridiplumi

A2F8E8FA-C66E-5874-A1EE-FF113ECF2F3E 10.3897/BDJ.13.e139552.suppl1 Supplementary material 1 Reference sequences used in Maximum Likelihood analysis Data type COI DNA sequence data File: oo_1240855.csv https://binary.pensoft.net/file/1240855 Lauren Rice, María Ana Tovar-Hernández, Christina Ellison, Craig Young

## Figures and Tables

**Table 1.** Specimen IDs and collection locations of *Seepicolaviridiplumi* gen. nov., sp. nov. used in genetic sequencing analysis along with their GenBank/BOLD accession numbers.

**Collection Location**	**Latitude**	**Longitude**	**Depth (m)**	**Sample ID**	**BOLD Process ID**	**GenBank Accession**
Bush Hill	27.78237117	-91.50830855	543	BHSa26	GMSE004-24	PP866051
				BHSa46	GMSE005-24	PP866050
				BHSa68	GMSE006-24	PP866049
				BHSa70	GMSE007-24	PP866048
Green Canyon 234	27.74614894	-91.22191704	538	GCSa44	GMSE008-24	PP866047
				GCSa47	GMSE009-24	PP866046
				GCSa49	GMSE010-24	PP866045

**Table 2.** Distinctive features of *Perkinsiana* Knight-Jones 1983, *Potamilla* Malmgren 1866, *Pseudopotamilla* Bush 1905 and *Seepicola* new genus.

Feature	*Perkinsianasensu* a variety of authors, as specified in each feature	Potamilla sensu Fitzhugh (1989)	*Pseudopotamilla* sensu Knight-Jones et al. (2017)	*Seepicola* new genus
Branchial crown	Symmetrical with most radioles same length (except 2-3 ventral-most developing radioles)(Tovar-Hernández, obs. pers.)	?	Asymmetrical with longest radioles dorsally	Symmetrical with all radioles same length (except 2-3 ventral-most developing radioles)
Dorsal and ventral branchial lobe flanges	Absent (Tovar-Hernández et al. 2012, Capa et al. 2019)	Absent	Present	Present
Palmate membrane	Absent (Fitzhugh 1989, Capa 2007); present or absent (Tovar-Hernández et al. 2012, Capa et al. 2019)	Present	Absent	Absent
Radiolar flanges	Absent (Fitzhugh 1989, Capa 2007); absent or present (Tovar-Hernández et al. 2012)	Absent	Absent	Absent
Radiolar eyes	Absent	Absent	Present, unpaired, compound in all radioles, except dorsalmost pair and some ventralmost radioles, usually limited to proximal half of radioles	Absent
Peristomial eyes	Present in juveniles (Capa et al. 2019)	?	May be present	Present
Radiolar appendages	Present (Fitzhugh 1989, Capa 2007, Tovar-Hernández et al. 2012, Capa et al. 2019)	Absent	Present	Present
Dorsal pinnular appendages	Present (Fitzhugh 1989); present or absent (Capa 2007, Tovar-Hernández et al. 2012, Capa et al. 2019)	Present	Present	Present
Ventral lips	Present (Fitzhugh 1989, Capa 2007, Tovar-Hernández et al. 2012, Capa et al. 2019)	Present	Present	Present
Parallel lamellae	Present (Fitzhugh 1989, Capa 2007, Tovar-Hernández et al. 2012, Capa et al. 2019)	Present	Present	Present
Ventral sacs	Absent (Capa 2007, Tovar-Hernández et al. 2012, Capa et al. 2019)	?	Present	Absent
Peristomial chambers	Never reported	Never reported	Never reported	Oval, translucent with a brown spot inside
Anterior margin of anterior peristomial ring	Low, of even height all around (Fitzhugh 1989, Capa 2007, Tovar-Hernández et al. 2012)	Low, of even height all around	Low, of even height all around	Low, of even height all around
